# Supplementary material for: Molecular basis for substrate specificity of the Phactr1/PP1 phosphatase holoenzyme
Source: eLife. 2020 Sep 25;9:e61509. doi: 10.7554/eLife.61509 (PMC7599070; doi:10.7554/eLife.61509)

Figure 3F (1); replicate (2)

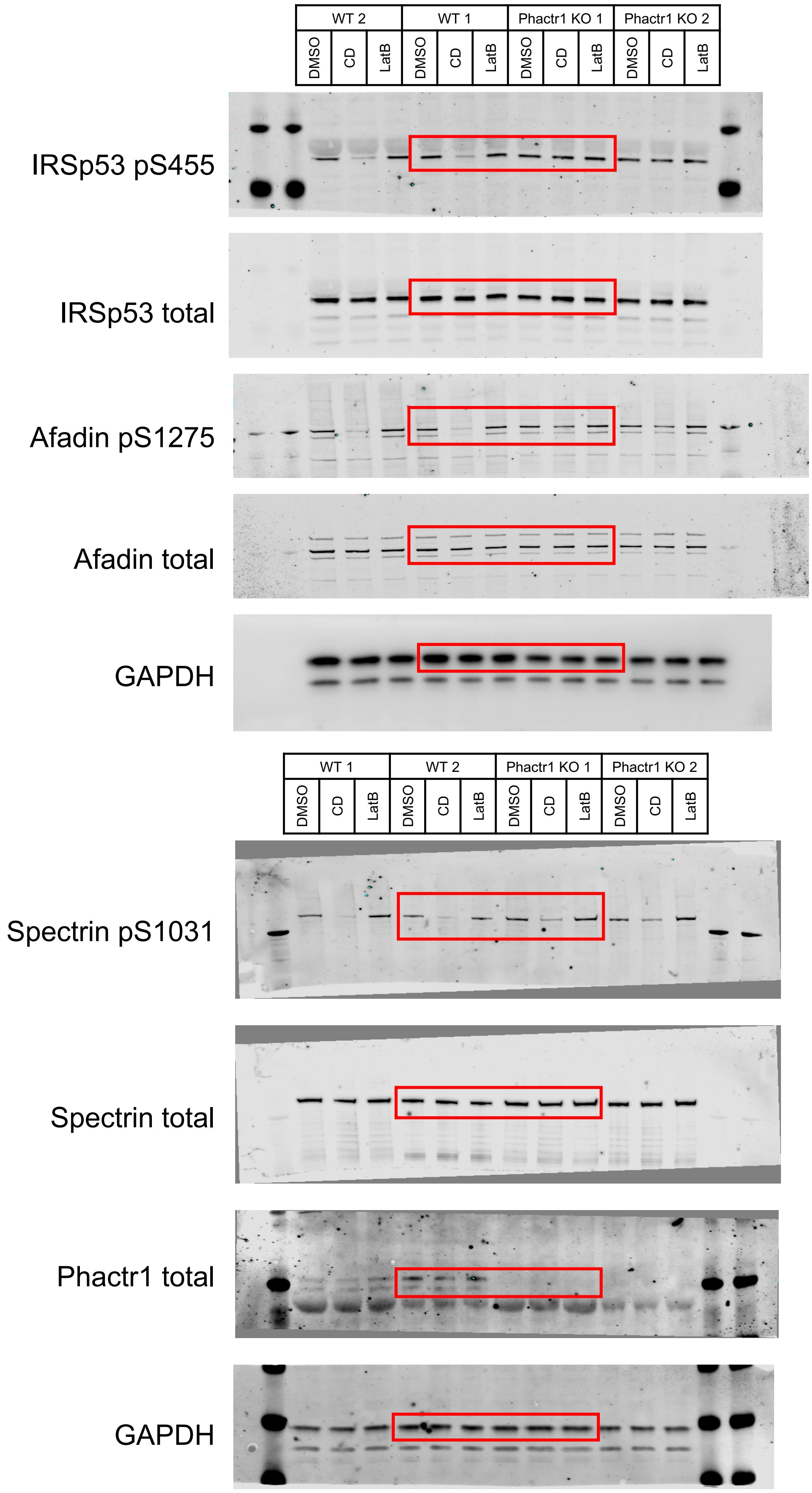

Figure 3 – Figure supplement 1, panel B

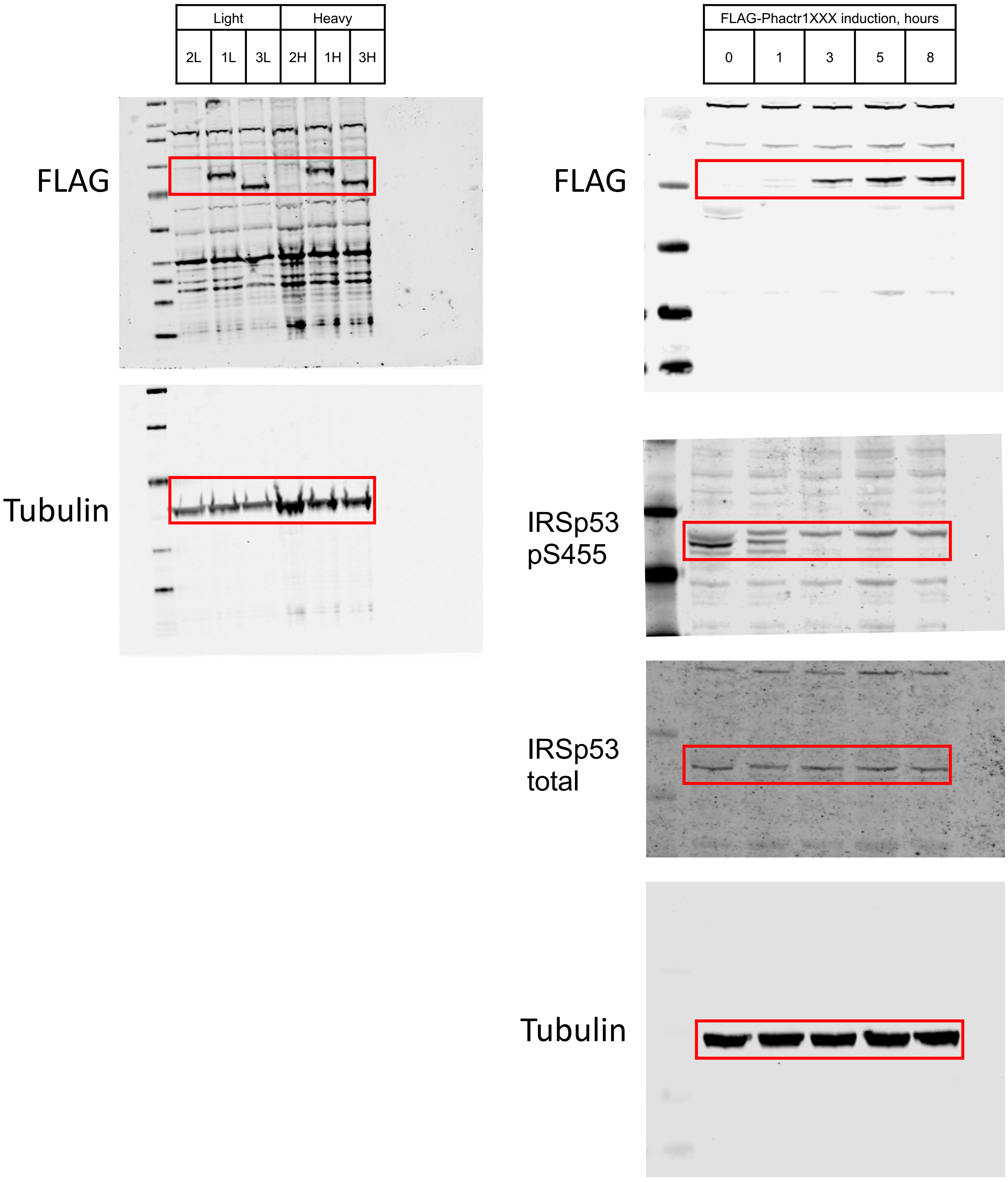

Figure 3 – Figure supplement 1, panels D, E

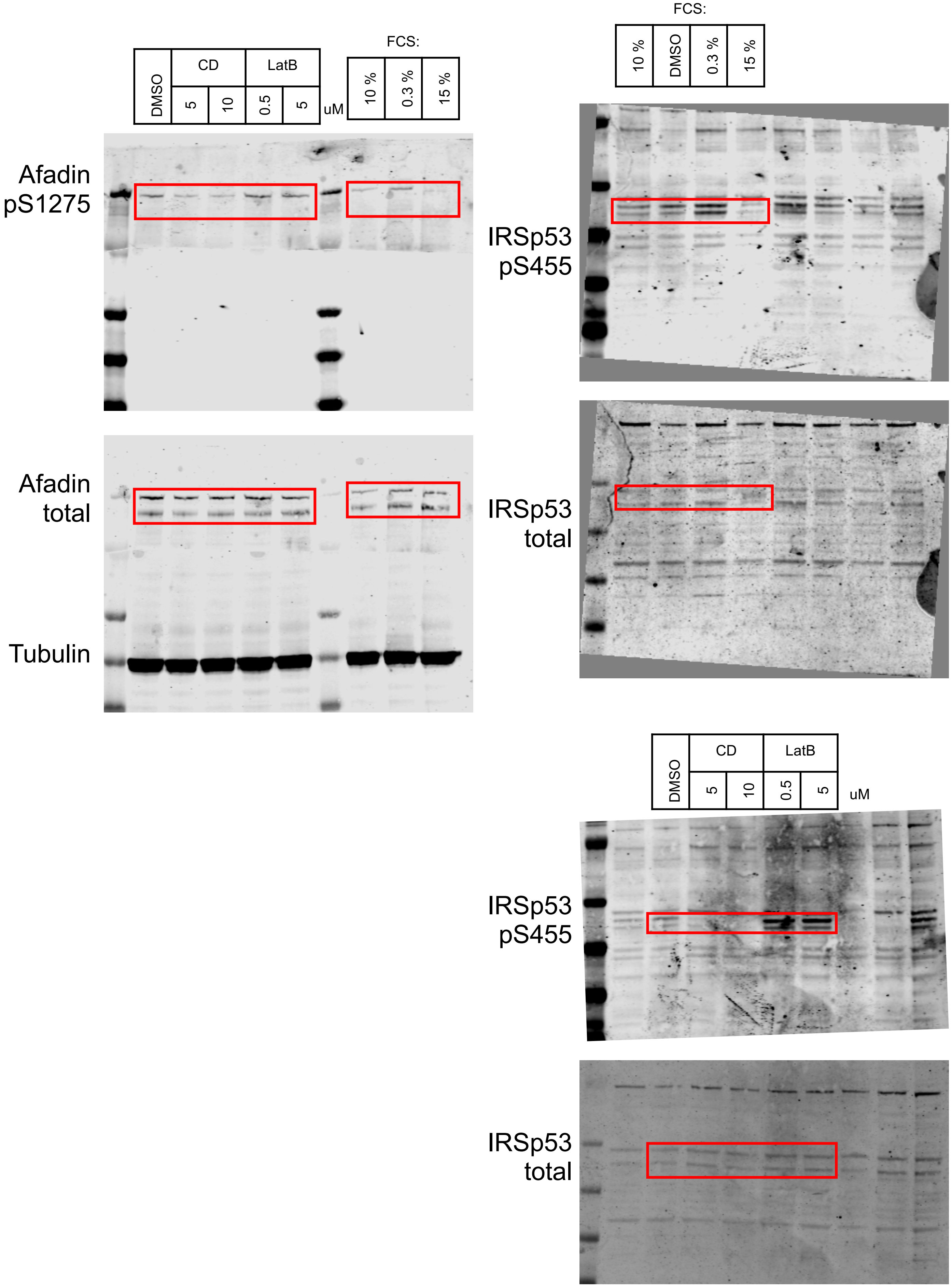

Figure 3 – Figure supplement 1, panels D, E (first replicates)

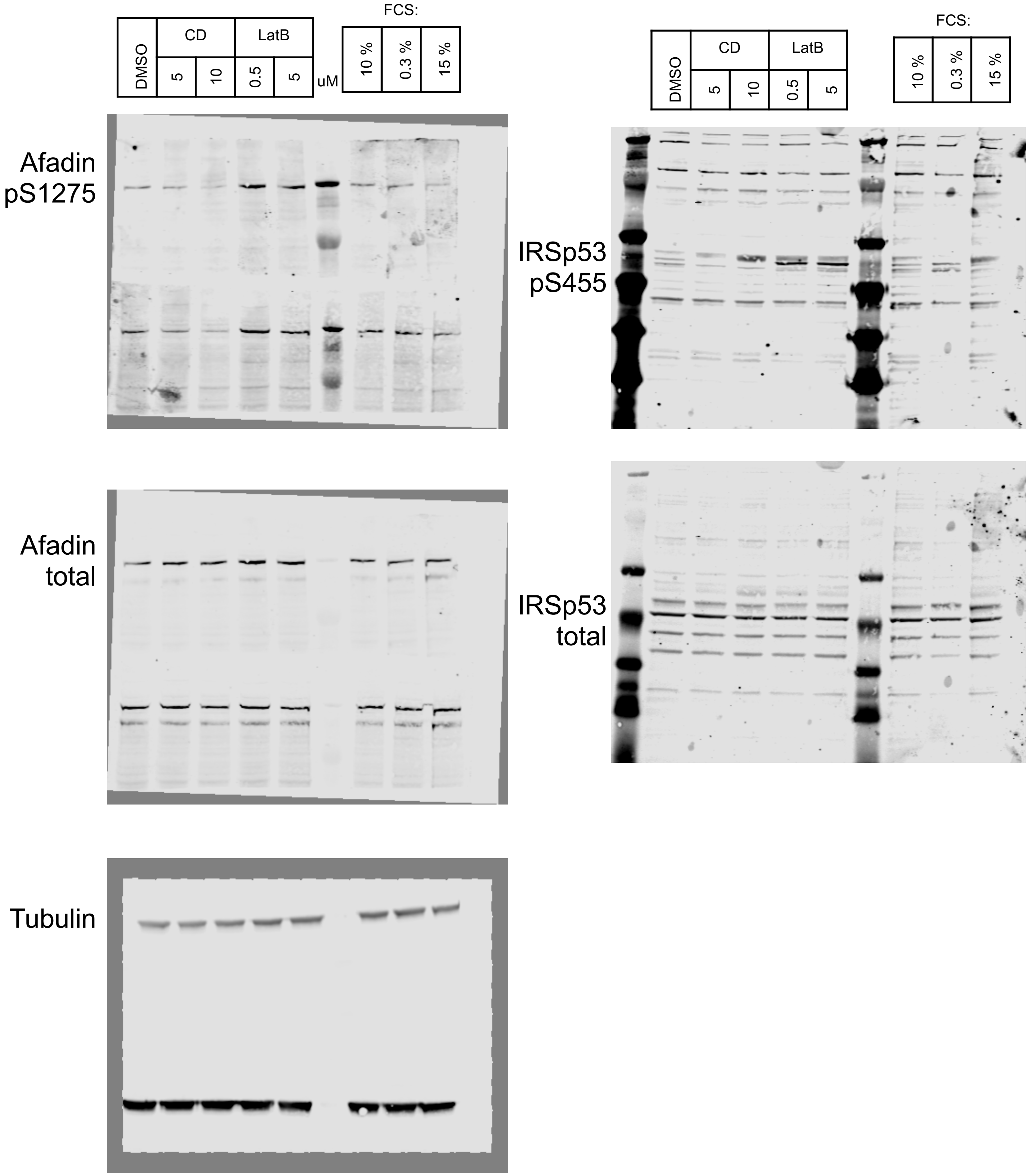

Figure 3 – Figure supplement 1, panels D, E (second replicates)

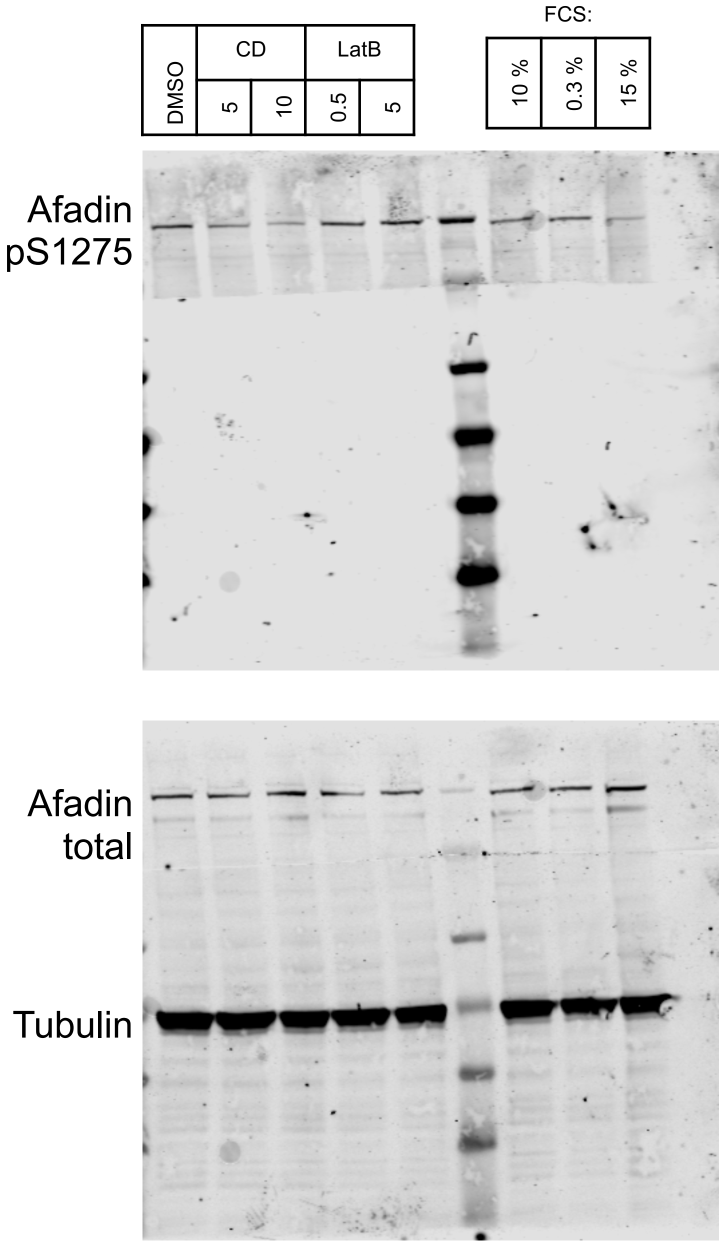

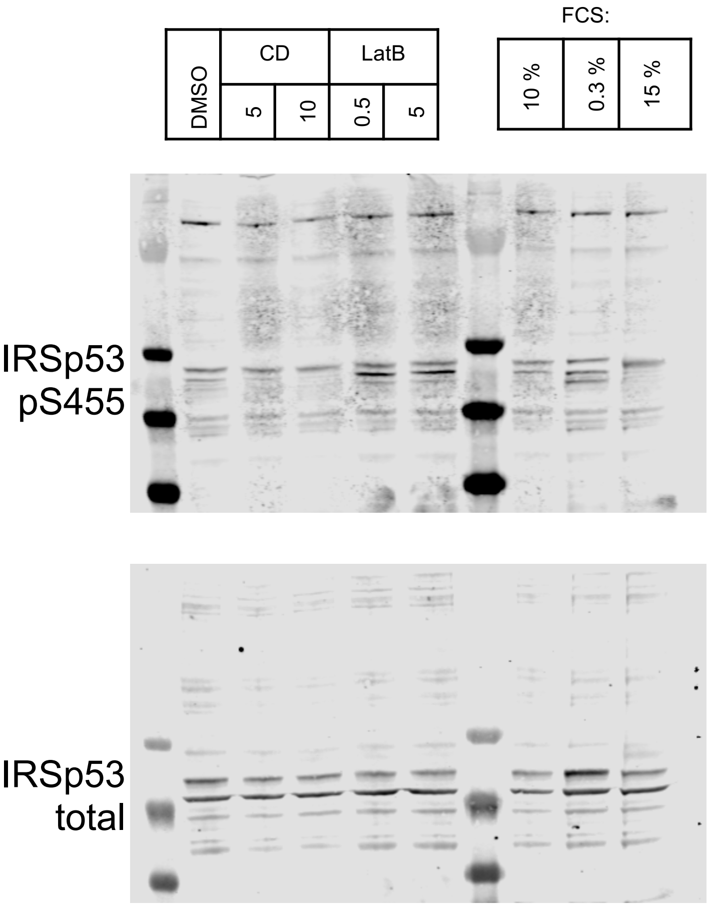

Figure 5C

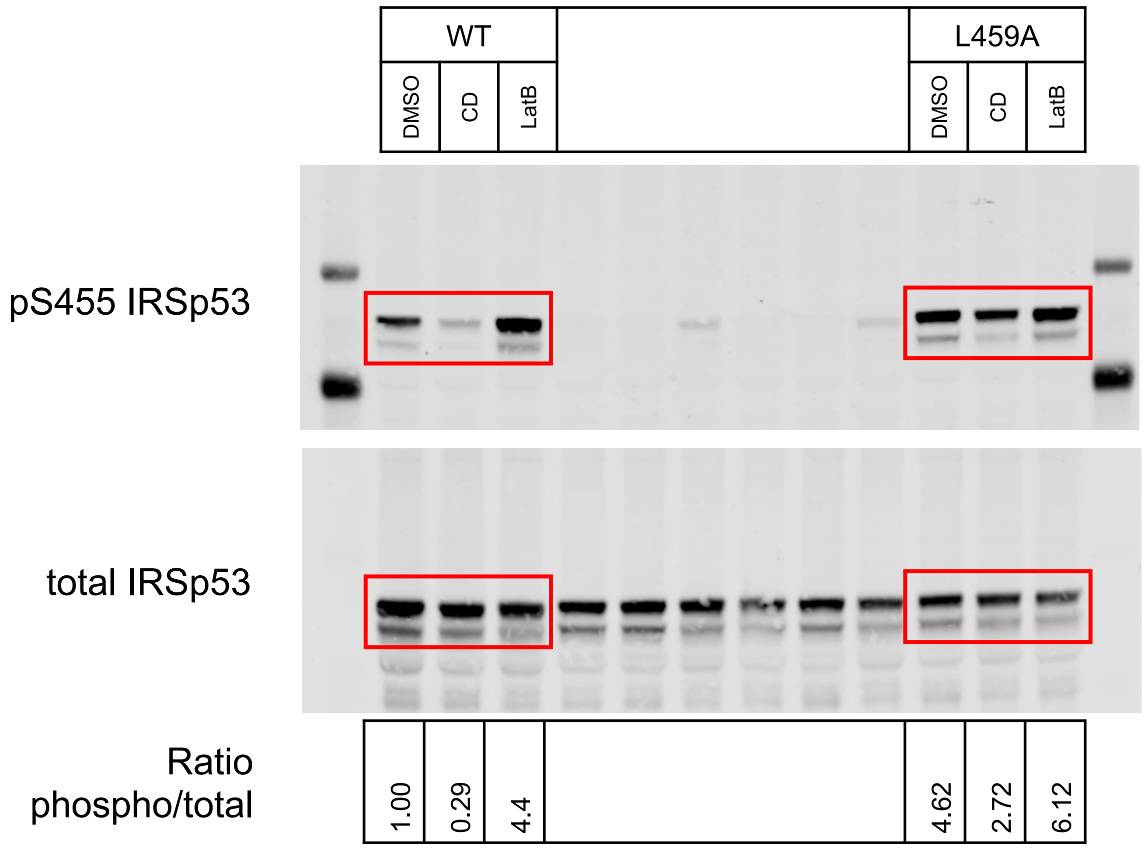

Figure 5C - replicate

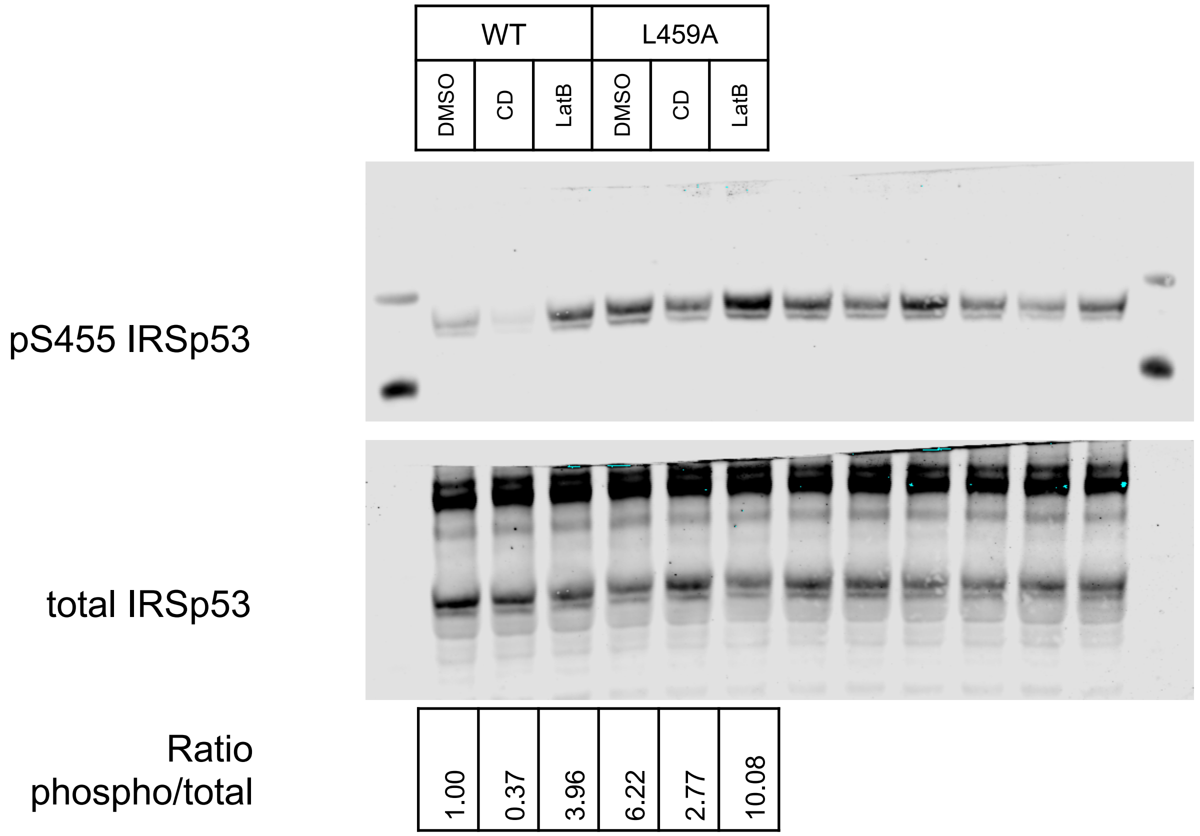

Supplement: Supplementary file 3. [file elife-61509-supp3.pdf]
