# Supplementary material for: Molecular basis for substrate specificity of the Phactr1/PP1 phosphatase holoenzyme
Source: eLife. 2020 Sep 25;9:e61509. doi: 10.7554/eLife.61509 (PMC7599070; doi:10.7554/eLife.61509)
Supplement: Supplementary file 5. [file elife-61509-supp5.docx]

**Supplementary File 5. Summary of modelled residues for the different structures**

| Structure | copy number | PP1 | Phactr1 | substrate |
| --- | --- | --- | --- | --- |
| Phactr1/PP1  (pH 8.5) | 1 | 7-299 | 513-580 |  |
|  | 2 | 7-299 | 513-580 |  |
|  | 3 | 7-299 | 516-580 |  |
|  | 4 | 7-299 | 512-580 |  |
|  | 5 | 7-299 | 516-580 |  |
|  | 6 | 7-299 | 512-580 |  |
| Phactr1/PP1  (pH 5.25) | 1 | 7-299 | 512-576 |  |
|  | 2 | 7-299 | 512-575 |  |
| Phactr1/PP1-IRSp53 | 1 | 7-299 | 513-580 | 449-462 |
|  | 2 | 7-299 | 513-580 | 452-464 |
| Phactr1/PP1-spectrin-αII | 1 | 7-298 | 515-580 | 1028-1039 |
|  | 2 | 7-298 | 516-580 | 1027-1037 |
| Phactr1/PP1-IRSp53-S455E | 1 | 7-299 | 513-580 | 449-462 |
|  | 2 | 7-299 | 513-580 | 449-464 |
| PP1-Phactr1(526-580) | 1 | 7-299 | 526-580 |  |
|  | 2 | 7-298 | 526-580 |  |
|  | 3 | 7-298 | 526-580 |  |
|  | 4 | 7-299 | 526-580 |  |
|  | 5 | 7-299 | 526-580 |  |
|  | 6 | 7-299 | 526-580 |  |
